# Botulinum Toxin A for Bladder Pain Syndrome/Interstitial Cystitis

**DOI:** 10.3390/toxins8070201

**Published:** 2016-07-01

**Authors:** Bin Chiu, Huai-Ching Tai, Shiu-Dong Chung, Lori A. Birder

**Affiliations:** 1 Division of Urology, Department of Surgery, Far Eastern Memorial Hospital, Ban Ciao, New Taipei City 220, Taiwan; femhuro@gmail.com; 2 Department of Urology, National Taiwan University Hospital, Taipei 100, Taiwan; taihuai48@hotmail.com; 3 Department of Medicine-Renal Electrolyte Division, University of Pittsburgh School of Medicine, Pittsburgh, PA 15261, USA; lbirder@pitt.edu; 4 Pharmacology & Chemical Biology, University of Pittsburgh School of Medicine, Pittsburgh, PA 15261, USA

**Keywords:** cystitis, botulinum toxin A, interstitial cystitis, intravesical injection

## Abstract

Botulinum neurotoxin A (BoNT-A), derived from Clostridium botulinum, has been used clinically for several diseases or syndrome including chronic migraine, spasticity, focal dystonia and other neuropathic pain. Chronic pelvic or bladder pain is the one of the core symptoms of bladder pain syndrome/interstitial cystitis (BPS/IC). However, in the field of urology, chronic bladder or pelvic pain is often difficult to eradicate by oral medications or bladder instillation therapy. We are looking for new treatment modality to improve bladder pain or associated urinary symptoms such as frequency and urgency for patients with BPS/IC. Recent studies investigating the mechanism of the antinociceptive effects of BoNT A suggest that it can inhibit the release of peripheral neurotransmitters and inflammatory mediators from sensory nerves. In this review, we will examine the evidence supporting the use of BoNTs in bladder pain from basic science models and review the clinical studies on therapeutic applications of BoNT for BPS/IC.

## 1. Introduction

Bladder pain syndrome/interstitial cystitis (BPS/IC) is a disease with clinical presentations of chronic pelvic/bladder pain, urinary frequency, and nocturia [1,2]. In the United States, it is estimated that about 6.5% or eight million women aged ≥18 years could be diagnosed as BPS/IC [3]. The possible mechanism of BPS/IC remains unclear, although several pathogeneses including neurogenic inflammation, autoimmune disorder, mast-cell activation and urothelial dysfunction [4,5,6,7,8] have been reported. Cystoscopy with hydrodistention under anesthesia and biopsy if indicated is the first choice for diagnosis, classification and treatment [1,2,9]. Among patients with BPS/IC, mucosal fissure with bleeding and Hunner’s ulcer are common findings after hydrodistension. According to guidelines of European Association of Urology, the available treatment options for patients with BPS/IC included the oral pentosan polysulfate sodium and intravesical Dimethyl sulfoxide (DMSO) which are both FDA-approved drugs [1,2]. Botulinum neurotoxins (BoNTs), which are produced by the bacterium Clostridium botulinum, have been widely applied medically since the first report of BoNTs for ocular strabismus in 1980 [10,11]. BoNT serotype A (BoNT-A) is the most widely used clinically, it can inhibit the release of neurotransmitters from nerve fibers and the urothelium [12,13,14]. Dykstra et al. firstly reported their experience of injections of BoNT-A into the external urethral sphincter to successfully treat detrusor sphincter dyssynergia of spinal cord injury patients [15]. Intravesical BoNT-A injection has been used in patients with BPS/IC since 2004. The pilot study reported that nine (69%) of 13 patients had subjective improvement after BoNT-A treatment [16]. However, recently, Gottsch et al. [16] investigated the efficacy of periurethral injection of 50 IU BoNTA in BPS/IC, and reported non-significant results on the main outcomes when compared with placebo. In the past decade, many studies have shown the efficacy of intravesical BoNT-A in BPS/IC though it has some side effects including urinary tract infection and urinary difficulty, and it seems to be a promising alternative treatment. In this review, we discuss the available mechanism and current medical evidence of BoNT-A for treating BPS/IC.

## 2. Mechanisms of BoNT-A

Internalization of BoNT/A begins with binding its ~100 kDa heavy chain to a specific receptor on the membrane of presynaptic neurons in the cholinergic nerve terminal. BoNT/A is then sequestered within a vesicle by endocytosis and undergoes a pH-dependent conformational change, leading to the translocation of its ~50 kDa light chain into the cytosol. After entering the cytosol, the light chain cleaves the SNAP-25 (synaptosome associated protein of molecular weight of 25 kDa), a subset of SNARE (soluble *N*-ethylmaleimide-sensitive fusion attachment protein receptor) proteins which mediate the fusion of the acetylcholine-containing vesicle with the presynaptic membrane. This interaction ultimately disrupts the release of neurotransmitter into the synapse, resulting in flaccid paralysis of the muscle [17]. There have been also several studies showing that BoNT/A inhibits the release of neurotransmitters that regulate pain and inflammation [18,19,20]. In an animal formalin-induced inflammatory pain model, the authors demonstrated the antinociceptive effects of BoNT-A [21]. Substance P and CGRP are neurotransmitters released by mainly C fibers, a primary nociceptive afferents of urinary bladder, Welch et al. found that BoNT can inhibit the release of substance P from cultured embryonic dorsal root ganglia neurons [22]. On the other hand, BoNT/A can diminish the amount of calcitonin gene-related peptide (CGRP) in rat trigeminal ganglia cells [23]. BoNT/A has been found to be able to reduce neurogenic inflammation, which is suggested to be associated with BPS/IC. In a capsaicin-induced prostatitis rat model, BoNT inhibited cyclooxygenase-2 expression [24]. Liu et al. demonstrated that BoNT-A injection to bladder can reduce the urine level of nerve growth factor, which is associated with neurogenic inflammation, by inhibiting the action of interleulkin-1, a proinflammatory cytokine [25]. In addition, BoNT can inhibit the delivery of the transient receptor potential vanilloid 1 (TRPV1) to neuron cell membranes [26].

TRPV1 has been identified to play an important role in the symptoms of urinary urgency, frequency and inflammatory pain in BPS/IC [27]. Liu et al. [27] showed that increased severity of bladder inflammation correlated with a higher expression of TRPV1-immunoreactive nerve fibers in BPS/IC patients’ bladders and correlated with clinical symptoms. The number of suburothelial afferents expressing TRPV1 was significantly reduced after BoNT-A treatment. The decreased levels of these sensory receptors are associated with a decreased sensation of urgency [27]. In addition, inhibition of purinergic P2X3 receptors on afferent terminals, which resulted in less ATP release from the urothelium can improve the painful sensations in BPS/IC. Apostolidis et al. analyzed the expression of P2X3 receptors in the bladder biopsy specimen of patients with detrusor overactivity after intradetrusor injection of BoNT-A demonstrated that P2X3-immunoreactive fibers were decreased significantly and the reduction of P2X3 expression significantly correlated with reduction of urgency episodes and urodynamic parameters [28]. Taken together, BoNT-A might improve the underlying neuroregulatory disorders of the urinary bladder in BPS/IC patients.

## 3. Clinical Evidence of Intravesical BoNT-A for BPS/IC

Several studies have supported the efficacy of BoNT-A in BPS/IC.

Smith et al. [16] first demonstrated the results of 200 IU BoNT-A trigonal and bladder base injection in treating BPS/IC in 13 patients and they showed a significant overall reduction in pain scores. Based on the previous study [29], which found most nociceptive bladder afferents are located in the trigone, Pinto et al. [29] performed trigonal injection with 100 IU BoNT-A in 26 women with refractory BPS/IC. The results demonstrated that pelvic pain, daytime and night-time voiding frequency, O’Leary-Sant Interstitial Cystitis Symptom Index (ICSI) and quality of life of all patients improved significantly. The maximal cystometric capacity increased and the efficacy remained in >50% of the patients for 9 months [30]. Giannantoni et al. [31] analyzed the efficacy of 200 IU suburothelial OnabotulinumtoxinA injection to trigone and bladder floor in 14 patients and they found that 12 patients (85.7%) had improvements in terms of daytime frequency, nocturia, visual analogue scale (VAS) score and increased cystometric capacity. Chung et al. reported their experience of 100 IU suburothelial BoNT-A injection immediately followed by cystoscopic hydrodistention in 67 refractory BPS/IC patients and the ICSI and Interstitial Cystitis Problem Index (ICPI), VAS score for pain, functional bladder capacity, and global response assessment (GRA) were all significantly improved compared to baseline data [32]. In another long term follow-up study in 15 patients, Onabotulinumtoxin-A at 200 IU submucosally injected to the bladder trigone and lateral walls, 13 patients (86.6%) reported subjective improvement regarding the mean VAS score, and daytime and nighttime urinary frequency [33]. However, at one year after treatment, pain recurred in all patients and nine patients suffered from dysuria 1 month after BoNT-A injection. A prospective, randomized study evaluated the responses of 15 patients and 29 received suburothelial injection with 200 IU or 100 IU of BoNT-A followed by cystoscopic hydrodistension 2 weeks later and 23 patients received the cystoscopic hydrodistension without BoNT-A injection [34]. All patients remained on baseline medications of pentosan polysulphate throughout the study and the results showed that intravesical injections of BoNT-A followed by hydrodistension can provide significantly better clinical results than hydrodistension alone with the BPS/IC symptom and VAS score decreased functional bladder capacity and cystometric bladder capacity increased. Among the total 44 patients in the BoNT-A group, 31 (71%) had clinically improvements at 6 months [34]. There was no efficacy difference between different BoNT-A doses treatment groups however the adverse events were more common in the group of 200 IU compared to 100 IU group. In a recent prospective, open labeled, randomized comparative study, patients with refractory BPS/IC received immediate injection or 1-month delayed injection of botulinum toxin type A, the results showed that the response rate was 73.5% at 1 month, 58.8% at 3 months, 38.2% at 6 months and 20.6% at 12 months [35]. Further multivariate analysis suggested that past exposure to hydrodistension more than three times is associated with a better outcome [35]. Another multicenter, randomized, double-blind, placebo-controlled trial in 60 patients with IC/BPS refractory to conventional treatment alsoindicated that hydrodistension plus suburothelial injections of BoNT-A 100 IU can significantly improve the VAS score and increase cystometric bladder capacity at week 8 after treatment. Bladder tissue obtained from 19 BPS/IC patients before BoNT-A injections and hydrodistension expressed significantly higher nerve growth factor (NGF) levels versus controls, and the level decreased after BoNT-A treatment especially in patients who responded clinically [36]. These findings implied that BoNT-A has an effect in reducing neuroplasticity of sensory fibers in the bladder, and supported the hypothesis of BPS/IC partly originated from neurogenic inflammation.

## 4. Conclusions

Intravesical BoNT-A injection is promising in the treatment of BPS/IC. BoNT-A can relieve pain, urgency and frequency for refractory BPS/IC though the success rates are variable. BoNT-A can also improve the functional bladder capacity and urodynamic parameters. However, there was no randomized placebo-control study to investigate the efficacy and improvement of quality of life of BoNT-A for BPS/IC patients. Further well-designed larger-scale randomized trials with placebo controls are warranted.
